# On the Response of a Herschel–Bulkley Fluid Due to a Moving Plate

**DOI:** 10.3390/polym14183890

**Published:** 2022-09-17

**Authors:** N’dri Arthur Konan, Eilis Rosenbaum, Mehrdad Massoudi

**Affiliations:** 1 U.S. Department of Energy, National Energy Technology Laboratory (NETL), 626 Cochrans Mill Road, Pittsburgh, PA 15236, USA; 2 NETL Support Contractor, 3610 Collins Ferry Road, Morgantown, WV 26507, USA

**Keywords:** boundary layer, Herschel–Bulkley fluid, yield stress, Carbopol, cement

## Abstract

In this paper, we study the boundary-layer flow of a Herschel–Bulkley fluid due to a moving plate; this problem has been experimentally investigated by others, where the fluid was assumed to be Carbopol, which has similar properties to cement. The computational fluid dynamics finite volume method from the open-source toolbox/library OpenFOAM is used on structured quad grids to solve the mass and the linear momentum conservation equations using the solver “*overInterDyMFoam*” customized with non-Newtonian viscosity libraries. The governing equations are solved numerically by using regularization methods in the context of the overset meshing technique. The results indicate that there is a good comparison between the experimental data and the simulations. The boundary layer thicknesses are predicted within the uncertainties of the measurements. The simulations indicate strong sensitivities to the rheological properties of the fluid.

## 1. Introduction

Constitutive modeling of complex fluids [1], sometimes referred to as non-linear fluids or non-Newtonian fluids, has received much attention in the literature [2,3,4]. Most of the naturally occurring and synthetic fluids are non-linear fluids, for example, polymer melts, suspensions, blood, slurries, drilling fluids, mud, etc., [5,6,7]. There are many empirical or semi-empirical constitutive equations that have been suggested for these fluids. Many non-linear constitutive relations have also been derived based on the techniques of continuum mechanics [8,9,10,11]. The non-linearities oftentimes appear due to higher gradient terms or time derivatives.

Cement and concrete are among two of the most interesting complex materials. In fact, at least since the publication of a paper by Rivlin & Ericksen [12], who discussed fluids of complexity *n* (see also Truesdell & Noll [13]), to the recently published book [14], the term ‘complex fluid’ refers, in general, to fluid-like materials whose response, namely the stress tensor, is ‘non-linear’ in some fashion. This non-linearity can manifest itself in a variety of forms such as memory effects, yield stress, creep or relaxation, normal–stress differences, etc., [15,16]. Cement has many applications, and it has been used in the oil and gas industries, where a cement slurry is pumped in the annulus space between the well casing and the geological formations surrounding the wellbore. This is carried out primarily to isolate the wellbore to keep fluids from migrating to other layers of the formation and secondly to prevent the corrosion and the eventual damage to the casing for the life of the well [17,18]. In time, the cement begins to harden. If the fluids from the surrounding formations penetrate the well, then disasters, both financial and operational, can occur, causing a shutdown and replacement of the cement. This unwanted phenomenon is known as ‘gas migration’ (see, e.g., [19]). In their powder forms, cement and concrete behave as bulk solids (granular materials); when mixed with water, initially they act as flowing suspensions (slurry) [20,21]; with chemical reactions and hydration occurring inside the suspension, the cement becomes a paste-like material (viscoelastic or viscoplastic) exhibiting yield stress and thixotropy [22,23]; and eventually, when it has hardened, the cement behaves as a poro-elastic material. Thus, cement can behave and can respond differently depending on the application and the conditions. When measuring its viscosity or its yield stress, cement generally behaves like a viscoplastic material.

In polymers, we can see a similar diversity. Agassant et al. [24] (p. xix) indicate that, in general, in polymer processing applications, one can distinguish three different stages: (1) the plastification (molten) stage where the polymer goes from a solid-like material, for example, powders or granular, to a fluid-like material, followed by (2) the molten polymer (see also [25]) being pushed or forced into molds or dies, and finally, (3) the stage where a final shape is given to the material, usually carried out via cooling. An excellent and early reference where the fundamentals of the modeling of these various stages in polymer processing are considered is the book by Middleman [26]. The Herschel–Bulkley (H-B) fluid model has been used in a variety of applications. Some recent applications are mentioned here. Ziaee at al. [27] studied colloidal-gas-aphron (CGA)-based fluids in drilling applications by modeling the fluid as a H–B fluid. In their study of solid-free polymer drilling fluid (SFPDF) with natural gas hydrates (NGH), Wang et al. [28], used the Herschel–Bulkley model. A new promising area for the application and use of polymeric gels seems to be in CO_2_ underground storage where supercritical gas tends to leak through microcracks in wellbores (see [29]), where in some cases cement slurries, used in oilfields, are too vicious and are not able to penetrate the cracks. Chauhan et al. [30] looked at the characteristics of gum karaya suspensions as a fracturing fluid and developed an empirical Herschel–Bulkley model capable of predicting the temperature and concentration sensitivity of the apparent viscosity. Zheng et al. [31] looked at the effects of temperature and the rheological impact of a commonly used drilling fluid polymer-treating agent used in the petroleum industries; they mention that the dispersion was reasonably described by a Herschel–Bulkley model. Millian et al. [32] studied the rheological behavior of gel polymer electrolytes (GPE) used as a suspending fluid in a zinc-slurry-air RFB by fitting their experimental data to the Herschel–Bulkley model.

In his pioneering work on flow of yield stress fluids, Oldroyd [33] proposed a plastic boundary-layer theory, defined as a region of sufficiently slow plastic flow characterized by a large Oldroyd (Od or Bingham) number and evolving in the limit of small Reynolds numbers. Oldroyd further argued that the thickness of such a plastic boundary layer is of the order of ${\mathrm{Od}}^{-1/2}d$ (where d is a characteristic length). The proposed theory relied upon a certain number of assumptions, especially at the boundary between the elastic and the plastic states of the material, in addition to the assumption of a constant positive sign of the velocity gradient inside the boundary layer. When such a slow steady plastic flow develops near infinite or semi-finite thin plates, Oldroyd discussed a case of constant thickness, as well as a case of a variable layer with a thickness ranging from zero at the leading edge to a finite value far away from the edge. He also derived expressions for both the velocity and the pressure distributions inside the boundary layer, which depend on the Oldroyd number.

Piau [34] discussed Oldroyd’s theory by pointing out certain inconsistencies buried in the approach; he mentioned five points. Essentially, these points can be summarized as the Dirichlet and the Neumann velocity boundary condition issues at the outer limit of the boundary layer where the transition occurs from a flowing material (a fluid) to an elastic material and at the wall. In addition, Piau pointed out that the assumptions on the pressure gradient and the symmetry conditions were not satisfied. Balmforth et al. [35] also claim that “Oldroyd’s analysis runs into difficulties when the boundary layer buffers a wall, being unable to satisfy all the boundary conditions and the continuity equation”. Piau revisited the theory and, in contrast to Oldroyd’s approach, the Bingham stress of the material’s plastic behavior was supplemented with the Hooke model for the linear elastic behavior that prevails in the outer unyielded regions. Piau [34] further identified and derived constant and variable lens-shaped boundary layer thicknesses and velocity distributions; he looked at the lower and the upper bound solutions consistent with the outer elastic region. The (viscous) drag forces acting on the plate in the context of these solutions, as derived by Piau, increase linearly with the yield stress and exhibits a relatively weak dependence on the Oldroyd (or the Bingham) number, which was assumed to be ‘large’ while the dependence of the drag forces was proportional to ${\mathrm{Od}}^{-1/2}$.

Piau & Debiane [36] extended this work to shear-thinning fluids in the context of a Herschel–Bulkley fluid with no-slip conditions at the walls. They showed that the boundary thickness along with the velocity distribution, as well as the (viscous) drag force acting on the plate, are explicitly functions of the power-law exponent. For instance, in the framework of the constant thickness model of the boundary layer, the slope of the velocity distribution is found to be determined by the power-law exponent. For the case of slip at the walls, Piau & Debiane [36] introduced a dimensionless number (ratio of the yield stress to the consistency index and the fluid velocity), which is a measure of the slip at the wall. In the context of the constant-boundary-thickness model, they found that slip at the wall reduces the viscous drag, while the fluid velocity inside the boundary layer increases with decreasing slip. Ahonguio et al. [37] experimentally investigated the influence of slip at the wall in the limit of non-inertial flow with relatively large Oldroyd numbers (varying from 16 to 40). These authors found that the slip velocity decreases with the Oldroyd number. The consequences are (i) a thinner boundary layer and (ii) a reduction in the drag, which is consistent with the slip at the wall described by Piau and Debiane [36], although the definition of the boundary layer thickness in Ahonguio et al. [37] is different from that used by Piau & Debiane. In another work, Ahonguio et al. [38] showed that their laboratory measurements of the drag coefficient for a Carbopol gel flowing past a thin fixed plate compared well with the Piau & Debiane model.

Balmforth et al. [39] revisited the derivation of Oldroyd’s theory by numerically studying flow past a thin plate. These authors also investigated a jet-like intrusion; these two examples, referred to as Oldroyd’s canonical problems, were meant to illustrate their approach. Most importantly, Balmforth et al. discussed that the magnitude of the small parameter (ϵ), associated with the re-scaling of the flow in the normal direction that sets both the thickness of the boundary layer and the angular velocity of the rotating plug, must be $Bi^{-1/2}$ (Bi being the Bingham number) in order to match the pressure within the viscoplastic boundary layer. These authors claim that there is “a missing ingredient in Piau’s boundary-layer scaling argument”.

In the context of a boundary layer developing away from rigid boundaries (referred to “remote boundary layers” by Oldroyd [33]), Chevalier et al. [40] discussed experiments of slow injections of a yield stress fluid into a stagnant fluid (the same fluid), by means of an extrusion syringe. They observed that the injected fluid penetrates as a solid-like block over the whole injection surface, while the large surrounding material remains at rest. Their study shows the existence of a thin boundary through which the motion of the injected material occurs. They also reported a decrease in that boundary layer with an increasing of the Bingham number. Chevalier et al. also looked at the data from Boujlel et al. [1] where a plate was slowly immersed into a bath of Carbopol gel at rest. As the fluid was stressed beyond the yield point, a thin layer around the plate developed. Boujlel et al. showed the measurements of the boundary layer size for various immersion velocities as well as the distribution of the velocity within the yielded region.

The response of a yield-stress fluid, for example, a Herschel–Bulkley fluid [41], due to the motion of a plate can provide useful information about the resistance (drag) to flow and how the plate can move in the yielded regions as opposed to the viscous regions of the flow. In a sense, this flow arrangement can be thought of as an idealization of the slow movement of a vane in a viscometer while measuring the yield stress of cement or a yield stress fluid [42]. Carbopol is a fluid which has been studied extensively and has similar properties to cement. In this paper, we present numerical solutions to the boundary-layer flow of a Herschel–Bulkley fluid showing a solid-like behavior away from the boundaries, which were reported by Boujlel et al. [1]. In Section 2, we provide a description of the problem while briefly mentioning the experimental investigation of Boujlel et al. [1], which is relevant to our work. This is followed by an overview of the mathematical model (in Section 3 and Section 4) used to describe the fluid and the flow conditions. The numerical method is presented in Section 5, and the results which are obtained using regularization approach in the context of the overset meshing technique are compared and discussed against the measurements of Boujlel et al. [1]. Finally, some conclusions and interesting points for future work are provided.

## 2. Problem Statement

**Figure 1:**
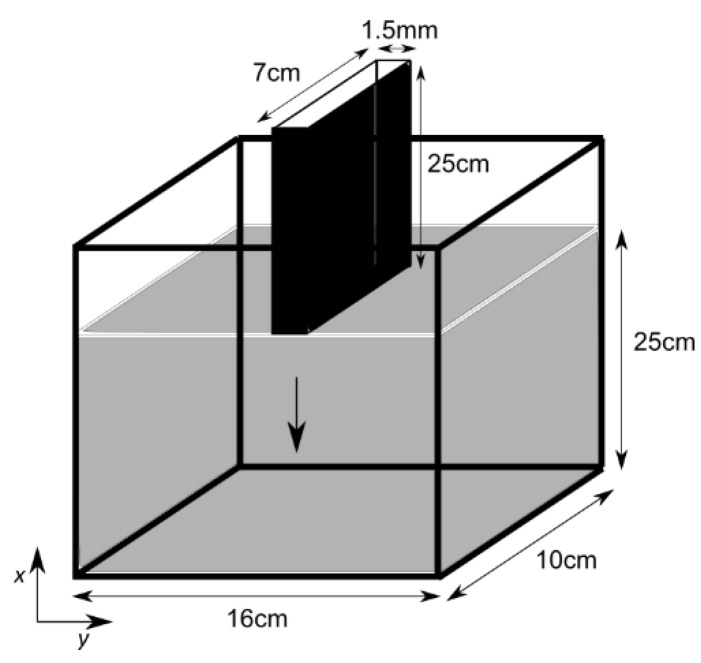
Sketch of the plate being immersed into the Carbopol fluid.

The experiments of Boujlel et al. [1] of a plate slowly being immersed in a yield stress fluid are numerically investigated here. The yield stress fluid, which is at rest in a parallel-piped-shaped container 10 cm wide, 25 cm high and 16 cm deep, is a solution of Carbopol in water with a concentration of 0.5%. The fluid is assumed to behave as a Herschel–Bulkley fluid, and its rheological properties are obtained from fitting of the rheometrical flow curves: the yield stress ($\tau_{0}$), the consistency (k), and the power-law exponent (n) are approximated as 59.5 Pa, 23.6 Pa.s^n^, and 0.38, respectively, for shear rates ranging between 10^−2^ s^−1^ and 10^2^ s^−1^. The plate is 25 cm long (l), 7 cm wide (w) with a thickness (d) 1.5 mm. The flow domain along with the immersed plate are sketched in Figure 1.

The flow conditions simulated in this work are summarized in Table 1. A total of three cases with different plate immersion velocity ($U_{p}$) are considered. As shown in Table 1, the flow conditions correspond to very small Reynolds numbers, usually associated with the Stokes regime (Re≪1). In the limit of such (creeping) flow conditions where the inertial effects are negligible, the flow depends on the Bingham number, which for these experimental conditions, indicates yield stress effects which dominate the viscous ones (by about 2 to 3 times). While the limit of small Reynolds numbers is attained, one may notice that the Bingham numbers are not as large as they are prescribed in the theories (see [33,34,36,39]). The Reynolds (Re) and the Bingham (Bi) numbers are defined below as (see the dimensionless form of the equation):

(1) Re=ρUp2/k(Up/d)n

(2) $$\mathrm{Bi}=\tau_{0}/k{\left(U_{p}/d\right)}^{n}$$

where $\tau_{0}$ is the yield stress, k the consistency, n the power-law exponent, ρ the density of the fluid, $U_{p}$ is a reference velocity, and d is a reference length. In their experiments, Boujlel et al. defined an observation window (of 5 cm × 6.5 cm) 5 cm below the Carbopol bath surface, where successive pictures were taken once the leading edge of the plate appeared in the window until it was immersed to a depth of 20 cm. The average velocity profile of the Carbopol as well as the boundary layer thickness discussed in the result sections below are extracted from these measurements at 15 cm above the leading edge of the plate. The boundary layer thickness is estimated as the flow region extent delineated in its upper bound by the constant fluid velocity.

As mentioned in the introduction, Piau & Debiane [36] showed that the boundary layer thickness (δ) can, in the context of the Herschel–Bulkley fluid, be approximated by a function which depends on the Oldroyd (or the Bingham) number:

(3) $$\delta ≅{d\;\mathrm{Bi}}^{-1/\left(1+n\right)}$$

## 3. Governing Equations

In this problem, we do not consider thermo-chemical or electromagnetic effects. Therefore, the governing equations of motion for a single component fluid include the conservation equations for mass, linear momentum, and angular momentum (see, e.g., [43]).

### 3.1. Conservation of Mass

(4) $$\frac{\partial \rho }{\partial t}+\mathrm{div}\left(\rho v\right)=0,$$

where ∂/∂t is the partial derivative with respect to time, div is the divergence operator, v is the velocity vector, and ρ is the density of the fluid. If the fluid is assumed to be incompressible, then it can only undergo isochoric (i.e., volume preserving) motions, so:

(5) div v=0

### 3.2. Conservation of Linear Momentum

(6) $$\rho \frac{dv}{dt}=\mathrm{div}T+\rho b,$$

where d/dt is the total time derivative given by d(.)/dt=∂(.)/∂t+[grad(.)]v and grad is the gradient operator, b is the body force vector, and T is the Cauchy stress tensor. 

### 3.3. Conservation of Angular Momentum

The conservation of the angular momentum indicates that the stress tensor is symmetric when there are no couple stresses, that is:

(7) $$T=T^{T}$$

Looking at the above equations, we can see that before we can solve any problems, we need a constitutive relation for T. In the next section, we provide a brief discussion of the stress tensor T used in this paper.

These conservation equations are supplemented with boundary and initial conditions, both at the walls and at the free-surface boundaries. The no-slip BC is prescribed at the plate and the walls such that:

(8) $$v=U_{p},\;\mathrm{at}\;\mathrm{the}\;\mathrm{plate}\;\mathrm{boundaries}$$

(9) v=0,  at the container’s walls

where $U_{p}$ is the plate immersion velocity. The Neumann zero gradient condition is imposed at the free surface for the velocity vector. For the initial conditions, since the fluid is initially at rest, we use:

(10) v(x,0)=0

## 4. Constitutive Relation for the Stress Tensor

For many complex fluids, yield stress is an important rheological parameter [44,45,46,47]. In the oil and gas industries, predicting the yield stress for cement slurries is also important [48]. Here, a cement slurry is pumped in the well and then it begins to hydrate quickly and develop strength [49]. The difficulties related to yield stress measurements have been discussed, for example in [50,51,52,53]. Coussot [53] and Coussot et al. [54] reviewed different methods for measuring the yield stress for thixotropic non-Newtonian fluids. Experimental measurements for the yield stress are usually conducted either by direct rheometric techniques or indirect techniques. For a detailed discussion related to cement applications, see the report by Tao et al. [55]. One of the disadvantages of the direct techniques is the wall-slip effects, which cause under-estimation of the yield stress [56,57]. One of the most widely used techniques to measure the yield stress is the vane method since there is no wall slip during the shearing process within the material [56,58,59,60]. Using the vane method [58,61], we can measure the peak torque–time response by rotating the vane immersed in the fluid. As mentioned in the Introduction section, the motion of a vane in the paste/suspension is of interest to us here.

In general, it can be assumed that the (Cauchy) stress tensor T for yield stress fluids, such as cement, can be defined as

(11) $$T=T_{y}+T_{v}$$

where $T_{y}$ is the yield stress tensor and $T_{v}$ is the viscous stress tensor. In general, for cement, the yield stress can be a function of many parameters, such as the volume fraction, *w*/*c*, etc.

(12) $$T_{y}=T_{y}\left(\phi ,\frac{w}{c},\ldots \right)$$

where ϕ is the volume fraction, and *w*/*c* is the water-to-cement ratio. In a recent review article, Tao et al. [62], proposed a very general constitutive relationship for $T_{v}$:

(13) $$T_{v}=-pI+\mu_{0}{\left(1-\frac{\phi }{\phi_{m}}\right)}^{-\beta }\left(1+\lambda^{n}\right){\left[1+\alpha \mathrm{tr}A_{1}{}^{2}\right]}^{m}A_{1}+\alpha_{1}A_{2}+\alpha_{2}A_{1}{}^{2}$$

(14) $$\frac{d\lambda }{dt}=\frac{1}{t_{0}}-\kappa \lambda \dot{\gamma }$$

where the kinematical tensors $A_{1}$ and $A_{2}$ are defined as:

(15) $$A_{1}=gradv+{\left(gradv\right)}^{T}$$

(16) $$A_{2}=\frac{dA_{1}}{dt}+A_{1}\left(\mathrm{grad}v\right)+{\left(gradv\right)}^{T}A_{1}$$

where p is the pressure, λ(t) is the structural parameter describing the degree of flocculation or aggregation. They used Krieger’s idea [63] for the volume fraction dependence of the viscosity, where $\mu_{0}$ is the (reference) coefficient of viscosity, tr is the trace operator, and m is the power law exponent, a measure of non-linearity of the fluid related to the shear-thinning effects (when *m* < 0) or shear-thickening effects (when *m* > 0). This model potentially is capable of exhibiting normal stress effects through the terms $\alpha_{1}$ and $\alpha_{2}$, thixotropy effects because of the presence of the structural parameter λ, shear-rate-dependent effects of the viscosity through the two parameters α and m (showing shear-thinning or shear-thickening effects), and the concentration dependency of viscosity through the two parameters $\phi_{m}$ and β. A simplified version Equation (13) was used in our earlier study [62].

For the yield stress part, historically, Oldroyd [33] derived a proper (frame invariant) 3D form for the Bingham fluid [64] by assuming that the material behaves as a linear elastic solid below the yield stress; he used the von Mises criterion for the yield surface. Thus:

(17) $$T=\left[\eta_{p}+\frac{\tau_{y}}{\sqrt{\frac{1}{2}{\mathrm{II}}_{A_{1}}}}\right]A_{1}\;\;\mathrm{when}\;\left[\frac{1}{2}T:T\right]\geq \tau_{y}^{2}$$

(18) $$T=GE\;\;\mathrm{when}\;\left[\frac{1}{2}T:T\right]<\tau_{y}^{2}$$

where *G* is the shear modulus, indicating that below the yield stress, the material behaves as a linear elastic solid, obeying the Hooke’s Law, and where E is the strain tensor and the second invariant of the tensor $A_{1}$ is:

(19) $${\mathrm{II}}_{A_{1}}\equiv A_{1}:A_{1}$$

As Denn [65] indicates, if the material is assumed to be inelastic prior to yielding, then G→∞, and Equation (18) is replaced by:

(20) $$A_{1}=0\;\;\mathrm{when}\;\left[\frac{1}{2}T:T\right]<\tau_{y}^{2}$$

Macosko [66] (p. 96) mentions that for many fluids with a yield stress, there is a lower *Newtonian* regime rather than a *Hookean* one, and thus one can use a two-viscosity (bi-viscous) model, such as:

(21) $$T=\eta_{p}A_{1}\;\mathrm{for}\;{\mathrm{II}}_{A_{1}}^{1/2}\leq {\dot{\gamma }}_{c}$$

(22) $$T=2\left[\frac{\tau_{y}}{{\left|{\mathrm{II}}_{A_{1}}\right|}^{1/2}}+K{\left|{\mathrm{II}}_{A_{1}}\right|}^{\frac{n-1}{2}}\right]A_{1}\;\mathrm{for}\;{\mathrm{II}}_{A_{1}}^{1/2}>{\dot{\gamma }}_{c}$$

where ${\dot{\gamma }}_{c}$ is the critical shear rate. In this paper, we use the Herschel–Bulkley model, and thixotropy is not considered. Thus, the stress in the fluid is given by:

(23) T=−pI+τ

where p is the pressure (the mean value of the stress tensor), I is the identity tensor, and τ is the stress tensor:

(24) $$\tau =\left[k{\left|{\mathrm{II}}_{A_{1}}\right|}^{\frac{n-1}{2}}+\frac{\tau_{0}}{{\left|{\mathrm{II}}_{A_{1}}\right|}^{1/2}}\right]A_{1}\;\mathrm{for}\;{\mathrm{II}}_{\tau }^{1/2}>\tau_{0}$$

(25) $$A_{1}=0\;\mathrm{for}\;{\mathrm{II}}_{\tau }^{1/2}\leq \tau_{0}$$

in which $\tau_{0}$ is the yield stress, k is the consistency index, and n is the power-law exponent, which measures of non-linearity of the fluid and is related to the shear-thinning effects (when n<1) or shear-thickening effects (when n>1). ${\mathrm{II}}_{\tau }$ and ${\mathrm{II}}_{A_{1}}$ are the second invariants of the stress tensor and of the kinematical tensor $A_{1}$. In Equation (24), the total contribution in the brackets, which defines the viscosity of the fluid, is the sum of the shear (viscous) ($\mu_{v}=k{\left|{\mathrm{II}}_{A_{1}}\right|}^{\left(n-1\right)/2}$) and the apparent ($\mu_{app}=\tau_{0}/{\left|{\mathrm{II}}_{A_{1}}\right|}^{1/2}$) viscosities.

In this paper, we ignore the micro-structure of the cement, i.e., the size and the shape of the particles, and the impact of the porosity and how the volume fraction affects the motion of the fluid. Thus, we represent the cement suspension as a viscoplastic fluid modeled as a Herschel–Bulkley fluid, given by Equations (23)–(25).

## 5. Numerical Approach

The computational fluid dynamics finite volume method from the open-source toolbox/library, OpenFOAM [67], is used on structured quad grids to solve the mass and the linear momentum conservation equations, using the solver “*overInterDyMFoam*” customized with non-Newtonian viscosity libraries. In our numerical scheme, we use the regularization methods. Indeed, to avoid the numerical implementation challenges associated with the discontinuity (singularity) in the stress tensor field between the unyielded and the yielded regions, the regularization method is used where the stress tensor τ is replaced with an ϵ-dependent small parameter such that:

(26) $$\tau_{\epsilon }=\eta_{\epsilon }\left({\left|{\mathrm{II}}_{2D}\right|}^{1/2}\right)A_{1}$$

where the ϵ-dependent viscosity $\eta_{\epsilon }$ is approximated in this work according to Papanastasiou [68] by:

(27) $$\eta_{\epsilon }\left({\left|{\mathrm{II}}_{A_{1}}\right|}^{1/2}\right)=k{\left|{\mathrm{II}}_{A_{1}}\right|}^{\left(n-1\right)/2}+\frac{\tau_{0}}{{\left|{\mathrm{II}}_{A_{1}}\right|}^{1/2}}\left[1-\exp\left(-\frac{{\left|{\mathrm{II}}_{A_{1}}\right|}^{1/2}}{\epsilon }\right)\right]$$

Two other commonly used regularization methods are also employed to study the sensitivity of the solution to such viscosity approximations; these two methods are the “simple” algebraic procedure (see e.g., [69]) and the approximation suggested by Bercovier & Engelman [70], given below, respectively:

(28) $$\eta_{\epsilon }\left({\left|{\mathrm{II}}_{A_{1}}\right|}^{1/2}\right)=k{\left|{\mathrm{II}}_{A_{1}}\right|}^{\left(n-1\right)/2}+\frac{\tau_{0}}{\epsilon +{\left|{\mathrm{II}}_{A_{1}}\right|}^{1/2}}$$

(29) $$\eta_{\epsilon }\left({\left|{\mathrm{II}}_{A_{1}}\right|}^{1/2}\right)=k{\left|{\mathrm{II}}_{A_{1}}\right|}^{\left(n-1\right)/2}+\frac{\tau_{0}}{{\left[\epsilon^{2}+\left|{\mathrm{II}}_{A_{1}}\right|\right]}^{1/2}}$$

A detailed examination of the convergence challenges and issues associated with the regularized solutions are discussed, for example, in Frigaard & Nouar [71] and Saramito & Wachs [72].

Substituting Equations (23)–(25) in Equation (6), we have the basic equations, which need to be solved numerically:

(30) div v=0

(31) $$\rho \left(\frac{\partial v}{\partial t}+\left[\mathrm{grad}v\right]v\right)=-\mathrm{grad}\;p+\mathrm{div}\left(\eta_{\epsilon }\left({\left|{\mathrm{II}}_{2D}\right|}^{1/2}\right)A_{1}\right)+\rho g$$

And the boundary conditions are,

- at the moving plate:

(32) $$v=U_{p},$$

(33) n·grad p=−ρn·[v·grad v] 

- at the container’s walls:

(34) v=0 

(35) n·grad p=0 

and the initial conditions are:

(36) v(x,0)=0,

(37) p(x,0)=0 

The dimensionless forms of the equations are presented in Appendix A.

**Figure 2:**
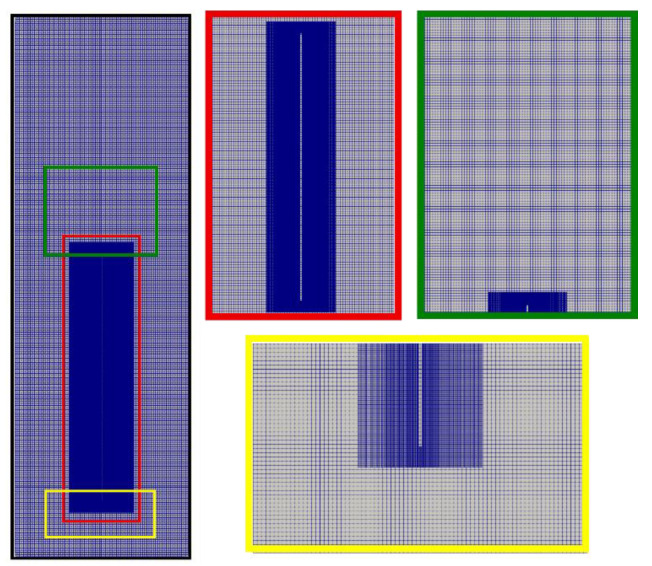
Overset grid of the flow domain.

The grid of the computational domain is generated relying upon the overset mesh technique, which in this work consists of a uniform grid (in each direction) of the background mesh of the entire flow domain, supplemented by a fine grid around the downward moving plate. With this fine (or overset) mesh, the grid in the normal direction to the plate is clustered using nonuniform spacings according to a geometric series with a rational stretching factor; this would better capture the gradients as the plate moves through the fluid. Figure 2 shows the background mesh, as well as the overset mesh which covers a region that extends over 3 cm at either side of the plate. Shown in Table 2 is the summary of the overset grid densities used to study the sensitivity of the solution to the mesh refinement.

The convective term in the momentum equations is discretized using the second order “linear” scheme. Spatial gradients are also discretized using the second order “linear” scheme (central differences with linear interpolation). The simulations are performed while discretizing the unsteady terms with a backward Eulerian scheme. The coupling between the background and the overset grids at these mesh boundaries is accomplished for the resolved fields (v,p) through a cell-volume-weighted interpolation scheme.

In the next section we discuss the results of our numerical simulations.

## 6. Results

### 6.1. Flow Visualization

**Figure 3:**
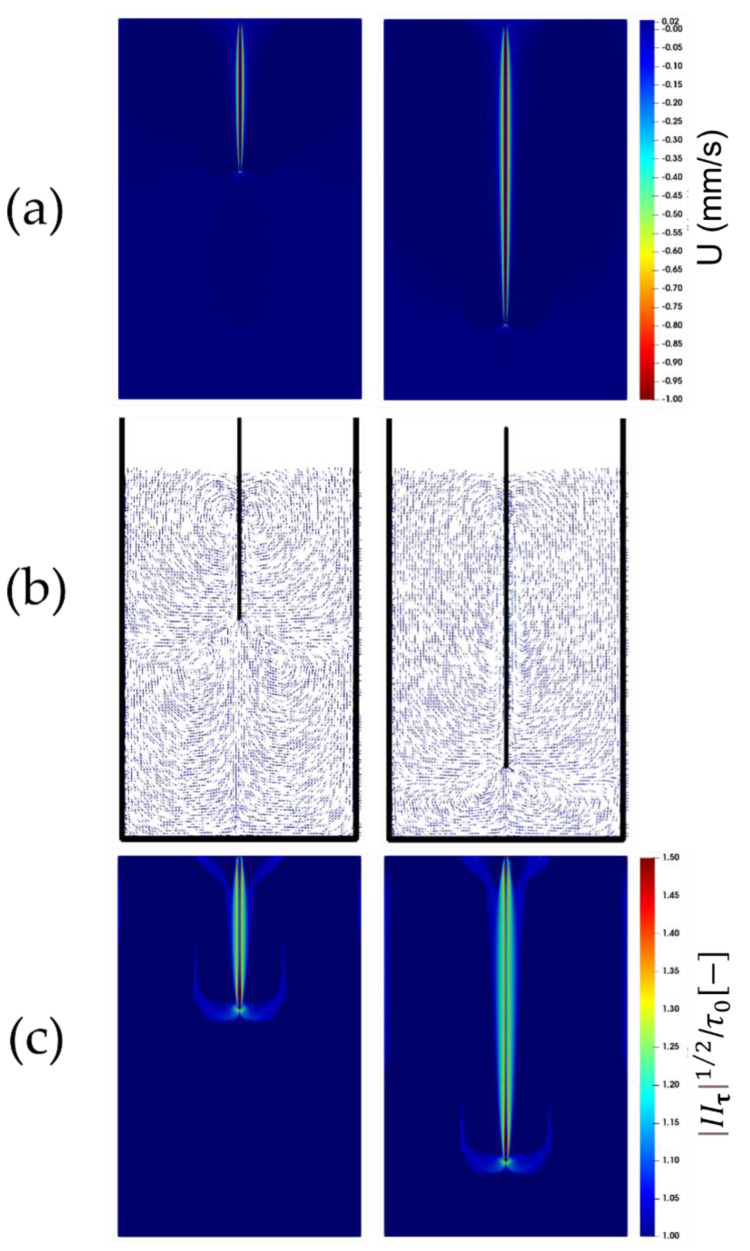
Snapshots of the flow for the plate immersion velocity of 1 mm/s. Parcels on the left-hand side show the immersion after 10 cm, while those on the right-hand side show the immersion after 20 cm. The top contour plots are (**a**) the fluid velocity, and (**c**) the bottom contours are the square root of the second invariant of the viscous stress tensor compared to the yield stress $\tau_{0}$. The middle vector (**b**) fields show the velocity vectors within the domain.

**Figure 4:**
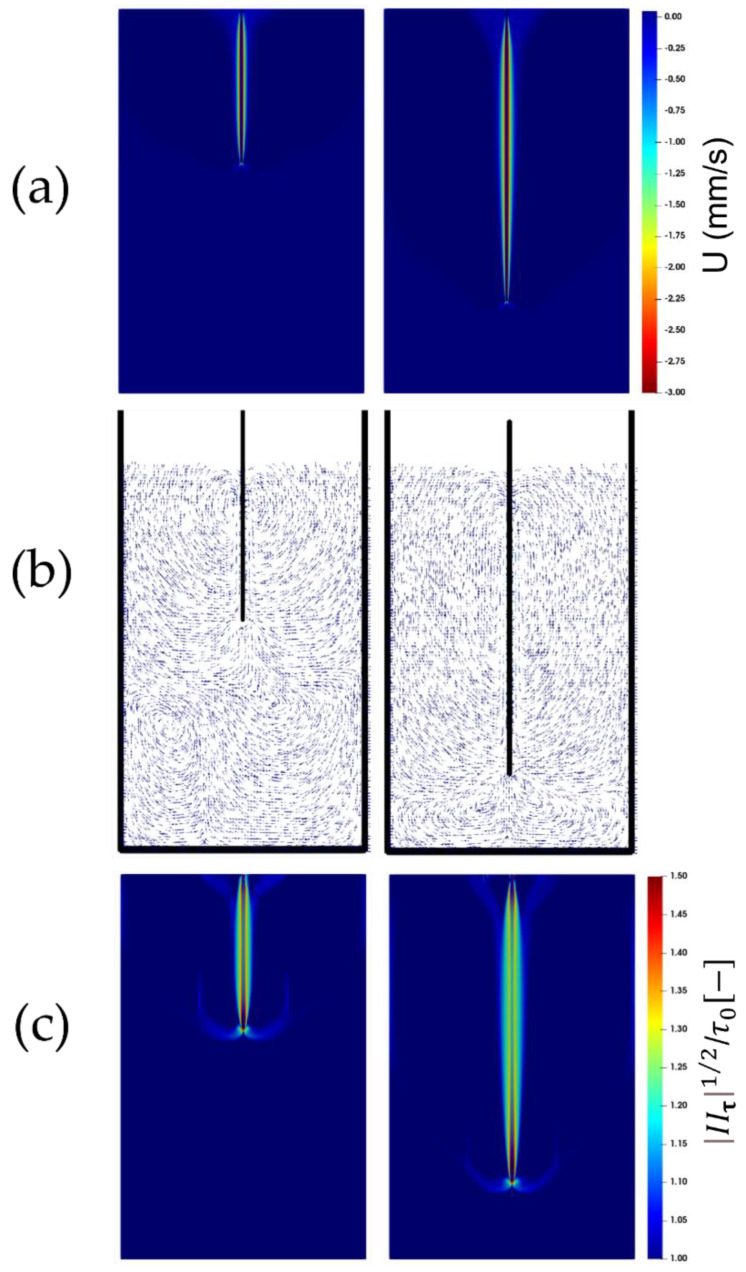
Snapshots of the flow for the plate immersion velocity of 3 mm/s. The top contour plots are (**a**) the fluid velocity, and (**c**) the bottom contours are the square root of the second invariant of the viscous stress tensor compared to the yield stress $\tau_{0}$. The middle vector (**b**) fields show the velocity vectors within the domain.

**Figure 5:**
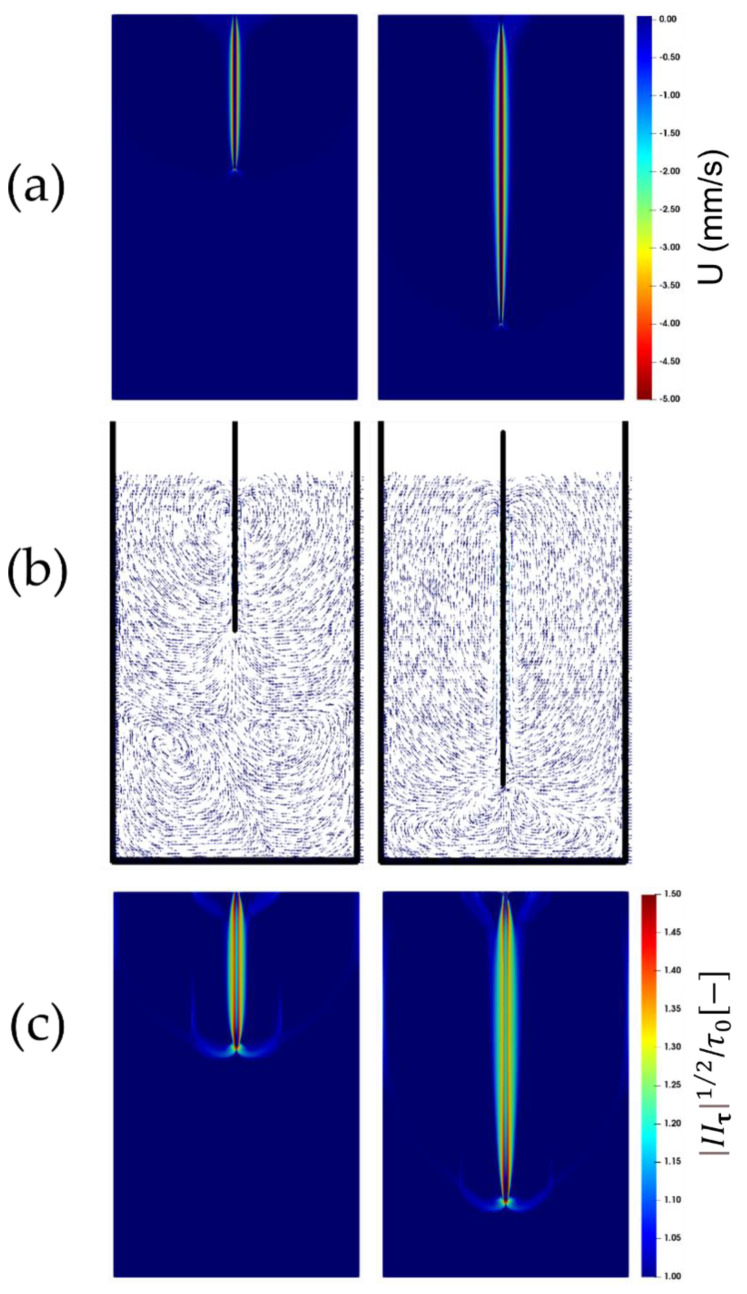
Snapshots of the flow for the plate immersion velocity of 5 mm/s. The top contour plots are (**a**) the fluid velocity, and (**c**) the bottom contours are the square root of the second invariant of the viscous stress tensor compared to the yield stress $\tau_{0}$. The middle vector (**b**) fields show the velocity vectors within the domain.

Figure 3a, Figure 4a and Figure 5a show the contour plots of the instantaneous (vertical) velocity of the fluid as the plate is being immersed. As seen, the fluid mainly moves within a narrow area around the plate. There are two recirculation zones below the plate’s leading edge, and the upward displacements at either side of the plate are readily apparent beyond this narrow area in the vicinity of the plate, where the fluid is dragged down by the plate (see Figure 3b, Figure 4b and Figure 5b). This flow pattern, which is consistent with the experimental observations, remains unchanged with an increase in the immersion velocity.

The square root of the second invariant of the viscous tensor (i.e., ${\left|{\mathrm{II}}_{\tau }\right|}^{1/2}$) plotted in Figure 3c, Figure 4c and Figure 5c, is compared against the yield stress $\tau_{0}$. It appears that the larger values of the second invariants of the viscous stress tensor are localized just around the plate. The fluid region, which is identified as the region where the second invariant exceeds the yield stress $\tau_{0}$, following the von Mises yield criterion, exhibits an anchor-like shape around the plate. This indicates that, in addition to the material, which behaves as a fluid within a small envelope surrounding the plate, there exists also a fluid-like region attached to the leading edge of the plate which extends at either side of the leading edge of the plate. This could have originated from the material deformation caused and sustained by the continuous penetration of the leading edge of the plate. Boujlel et al. [1] reported that although some material was liquefied just below the leading edge of the plate, most of the material was liquefied around the leading edge. Furthermore, two narrow fluid regions also appear at the walls of the container.

**Figure 6:**
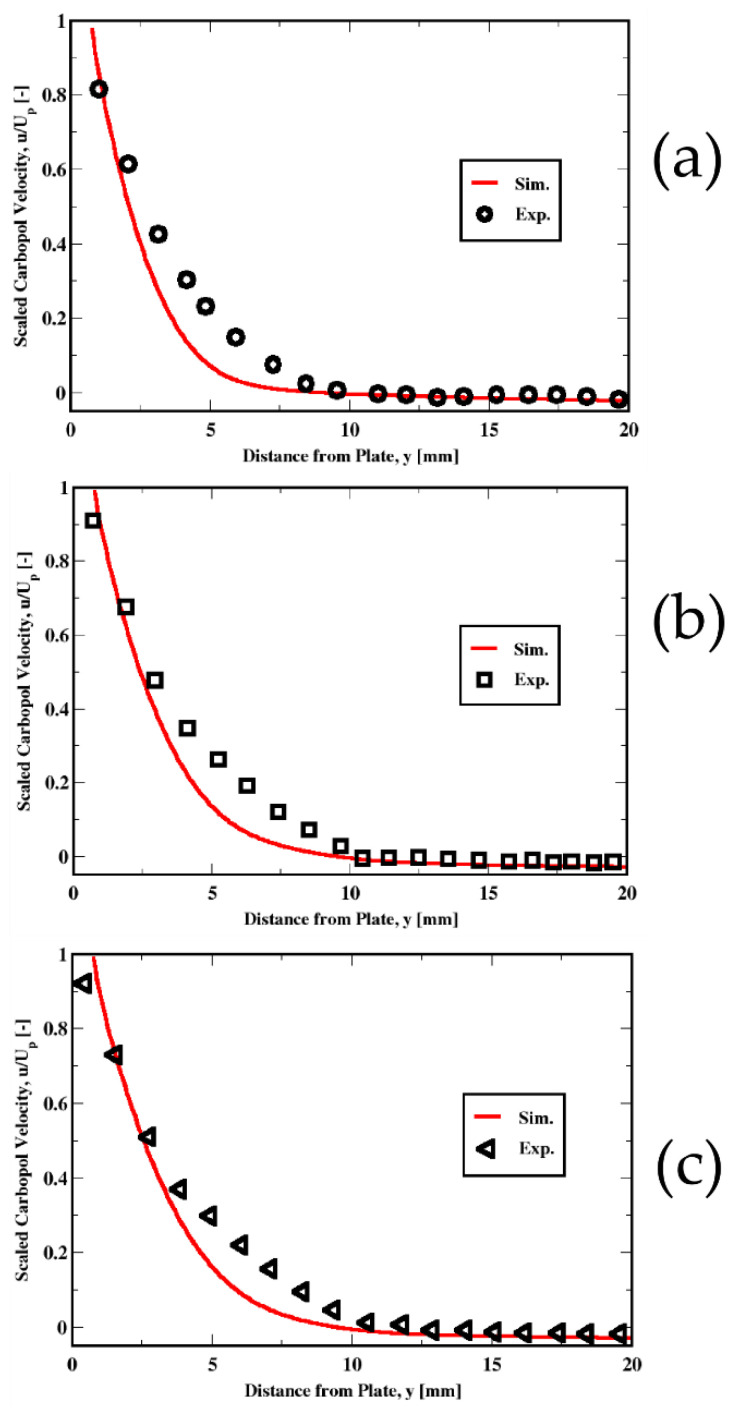
Distribution of the velocity of the Carbopol fluid. Plots from top (**a**), middle (**b**), and bottom (**c**) refer to the immersion velocities of 1 mm/s, 3 mm/s, and 5 mm/s, respectively.

Figure 6a–c compares the numerical predictions of the fluid velocity for the three different immersion velocities of the plate. These comparisons show that, away from the plate, within the previously identified solid-like region, the upward velocities of the fluid are accurately captured. Near the plate, where the Carbopol behaves like a liquid, the predictions are satisfactory as well. However, a slight mismatch of the profiles against the measurements occurs as the solid-like behavior region is approached. The reason is unclear. However, we can speculate that the solid–fluid transition, where both the solid and the liquid regimes could potentially co-exist, may have not been adequately captured in the simulations. Putz & Burghelea [73] reported from their experimental observations that such a transition is characterized by a competition between destruction and reformation of the gel.

**Figure 7:**
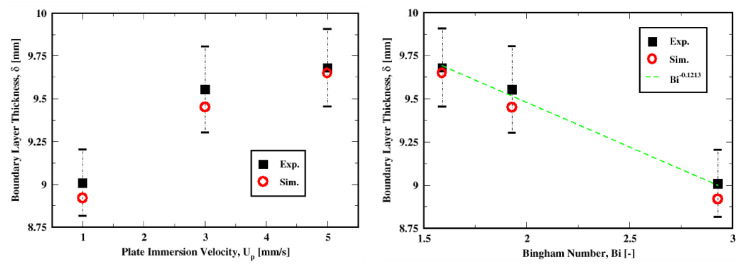
Thickness of the boundary layer with respect to the immersion velocity of the plate (**left**) and the Bingham number (**right**).

The boundary-layer thickness, measured as the extent of the yielded region adjacent to the plate, is plotted for the three velocities in Figure 7. The plot shows that the variation in the yielded region with the increase in the plate velocity is adequately predicted both in trend and quantitatively; the maximum relative error over these three conditions is only about 1.07%. The predictions are indeed within the uncertainties of the experimental data as shown by the error bars.

We can see from Figure 7 that an increase in the Bingham number results in a decrease in the boundary layer thickness. This suggests that for creeping flows, where the yield stress dominates over the viscous stress, the boundary layer developing around the moving plate will exhibit a negative correlation dependence on the Bingham number. This is consistent with the boundary layer theory proposed by Piau & Debiane [36], which is an improvement and extension to the Herschel–Bulkley model of Oldroyd’s theory [33]. Indeed, Piau & Debiane showed that the boundary layer thickness scales as $Bi^{-1/\left(1+n\right)}$, which with the current rheological properties results in $Bi^{-0.7246}$ (given that n=0.38). However, the experimental data (or at least the subset investigated in this work), indicate that the thickness scaled as $Bi^{-0.1213}$.

### 6.2. Mesh and Regularization Sensitivities

**Figure 8:**
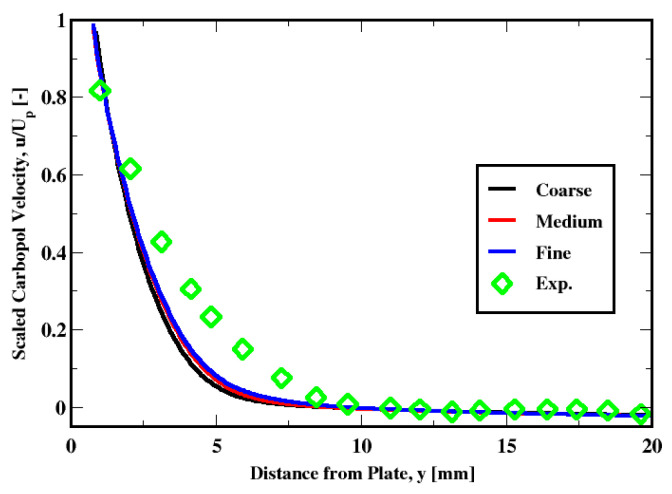
Sensitivity of the velocity distribution to the mesh refinement.

The sensitivity of the solution to the mesh and the regularization method are examined below for the plate velocity of 1 mm/s. The sensitivity to the mesh resolution is performed using Papanastasiou’s approximation (with ϵ = 10^−5^). The mesh densities along with the resolutions are summarized in Table 2. Figure 8 compares the velocity profiles for the three grids against the measurements. It is apparent that the medium-grid solution does quite well with the fine-grid prediction, while the coarse-grid one slightly deviates from those two solutions. Furthermore, it can be seen from the fine-grid solution that the slight mismatch remains despite the refinement, especially in the vicinity of the plate. As summarized in Table 3, the predicted boundary layer thickness with respect to different grids are within the measurement uncertainties. The prediction with the medium grid compares well with the fine grid predictions and the slight mismatch in the velocity profile marginally reflects grid resolution issues.

**Figure 9:**
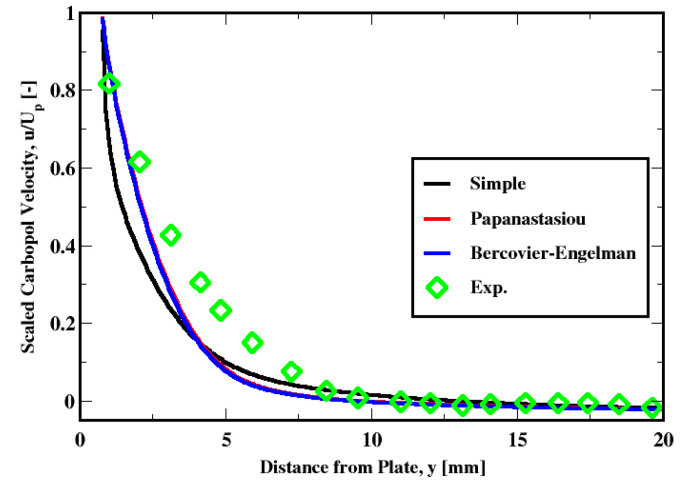
Sensitivity of the velocity distribution to the regularization model.

The influence of the regularization method is shown in Figure 9. This plot shows that there is little difference between the Bercovier–Engelman and the Papanastasiou methods. The boundary layer thickness prediction compared in Table 3 shows that the difference is very small. As for the “simple” regularization approach, the velocity profile seems to be less accurately predicted; the same observation holds for the thickness of the boundary layer (see Table 3).

### 6.3. Results for Different Fluid Properties

Here, we look at the difference between the numerical predictions of the velocity inside the boundary layer obtained by prescribing the thickness of the boundary layer (δ), and the velocity distribution measured by Boujlel et al. (2012). This velocity distribution is a function of the boundary layer thickness (δ) (see [1,36]):

(38) $$u=U_{p}\left[1-{\left(1-\frac{y}{\delta }\right)}^{1+1/n}\right],\;\;0<y<\delta \;$$

where $U_{p}$ is the plate velocity and n is the power-law exponent of the Herschel–Bulkley fluid model.

**Figure 10:**
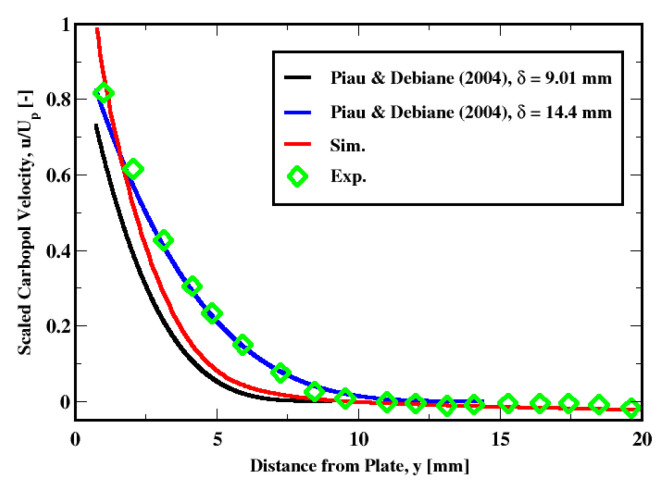
Predictions of the velocity distribution using Piau and Debiane (2004) model for a prescribed boundary layer thickness.

In Figure 10, we plot the velocity distribution using the measured thickness of δ=9.01 mm and δ=14.4 mm proposed by Boujlel et al. The plot prediction using the measured thickness exhibits a less satisfactory agreement contrary to the larger boundary layer thickness for which a very good agreement is found. Since the only difference is due to a different value of the boundary layer thickness, which depends on the Bingham number, we think it would be interesting to look at different values for the properties of the fluid and see the impact on the results.

Although Boujlel et al. [1] carefully prepared and performed the measurements, they did not mention uncertainties in their measurements. Piau [74] argued from an extensive literature review that often the rheometry of Carbopol aqueous gels is not without measurement difficulties.

We now look at the boundary-layer flow for different properties of a yield-stress fluid modeled as a Herschel–Bulkley fluid. The results for the velocity profiles show a systematic slight mismatch of the predictions against the measurements in the yielded region behaving as a solid-like material. Table 4 lists the different rheological properties which we use in our simulations.

**Figure 11:**
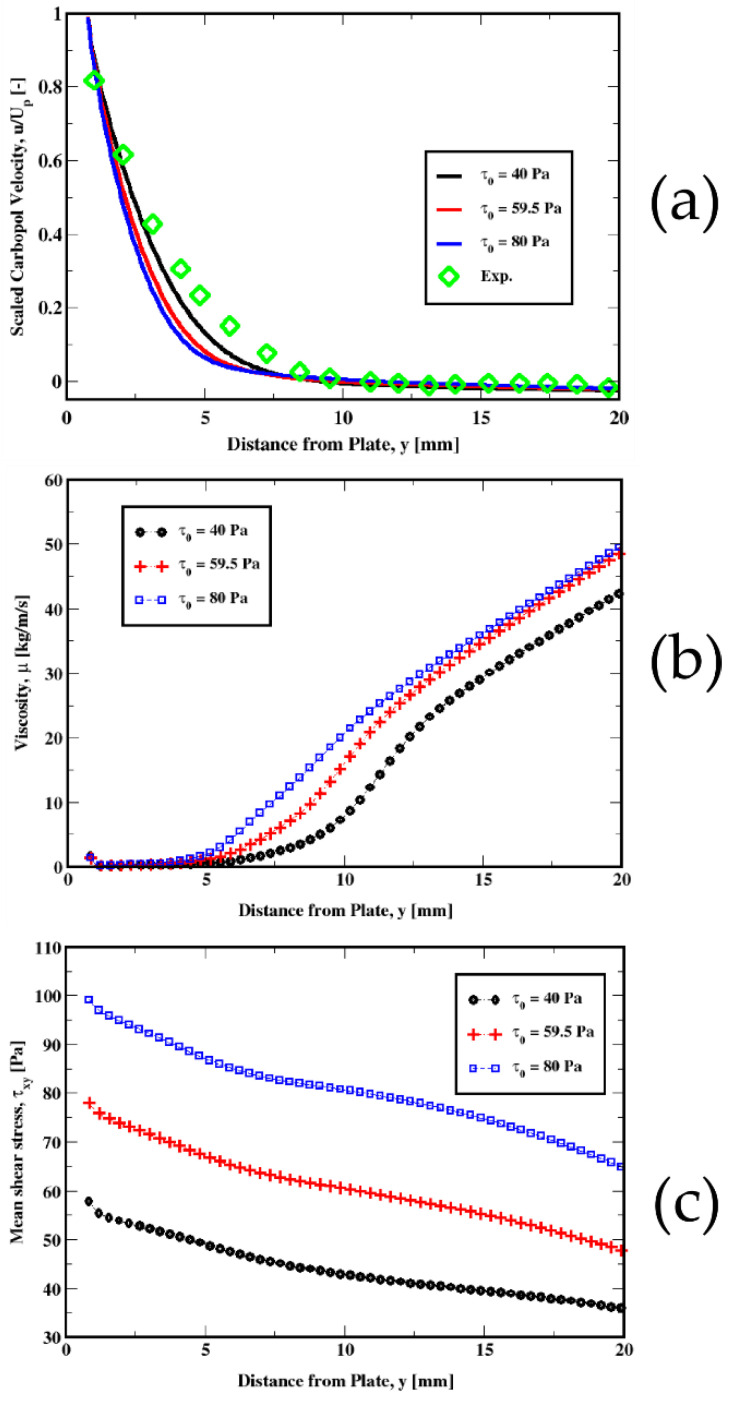
Influence of the yield stress on the distribution of the velocity (top plot) (**a**) for the plate immersion velocity of 1 mm/s. The middle (**b**) and the bottom (**c**) plots represent the variations in the kinematic viscosity and the mean shear stress, respectively.

**Figure 12:**
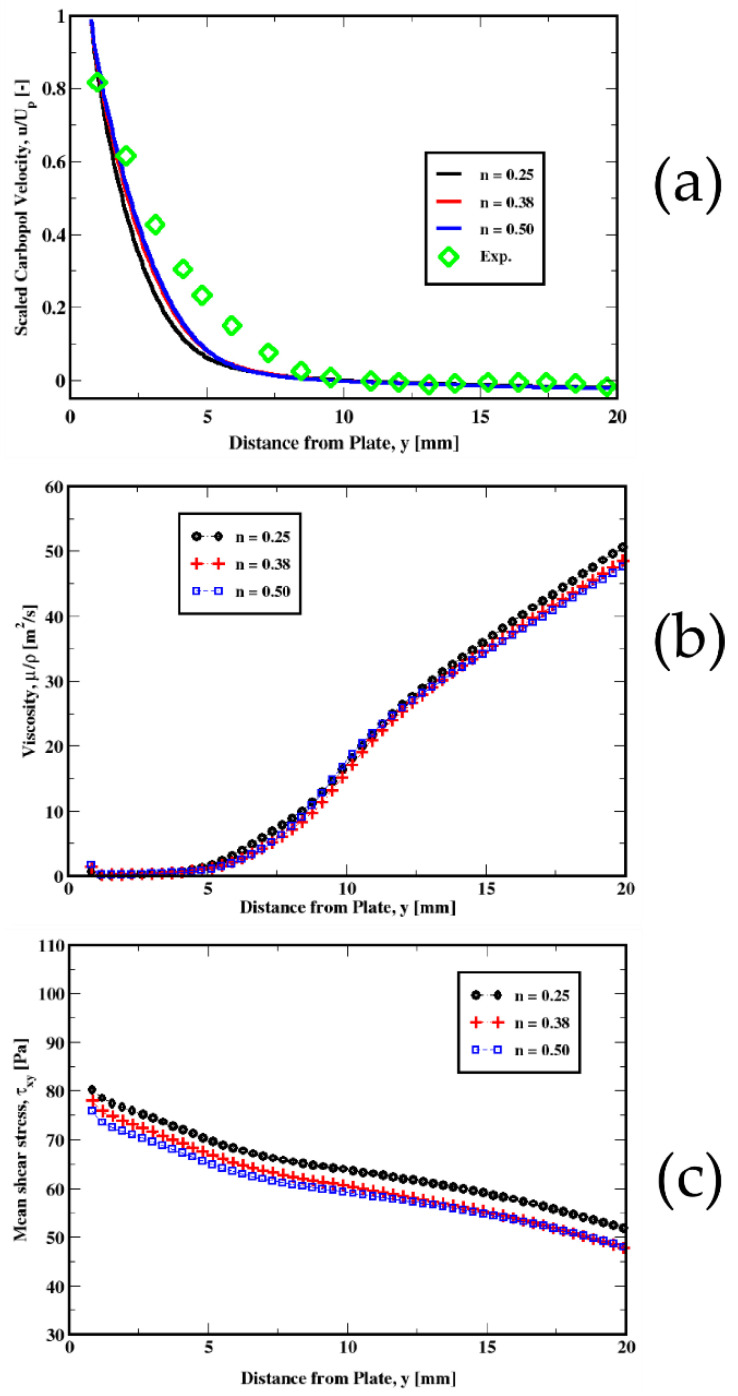
Influence of the power-law exponent on the distribution of the velocity of the fluid (**a**) (top plot) for the plate immersion velocity of 1 mm/s. The middle (**b**) and the bottom(**c**) plots represent the variation in the kinematic viscosity and the mean shear stress, respectively.

**Figure 13:**
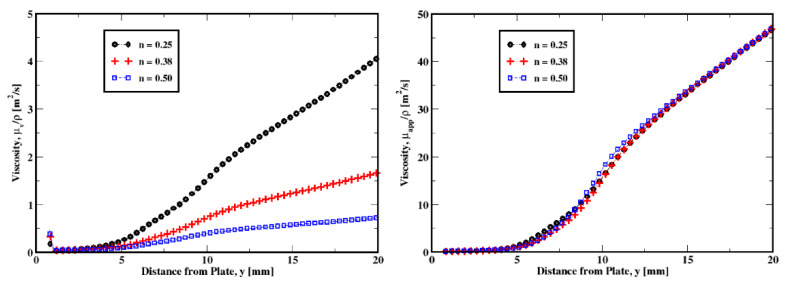
Influence of the power-law exponent on the viscous (**left** plot) and the apparent (**right** plot) viscosities for the plate immersion velocity of 1 mm/s.

**Figure 14:**
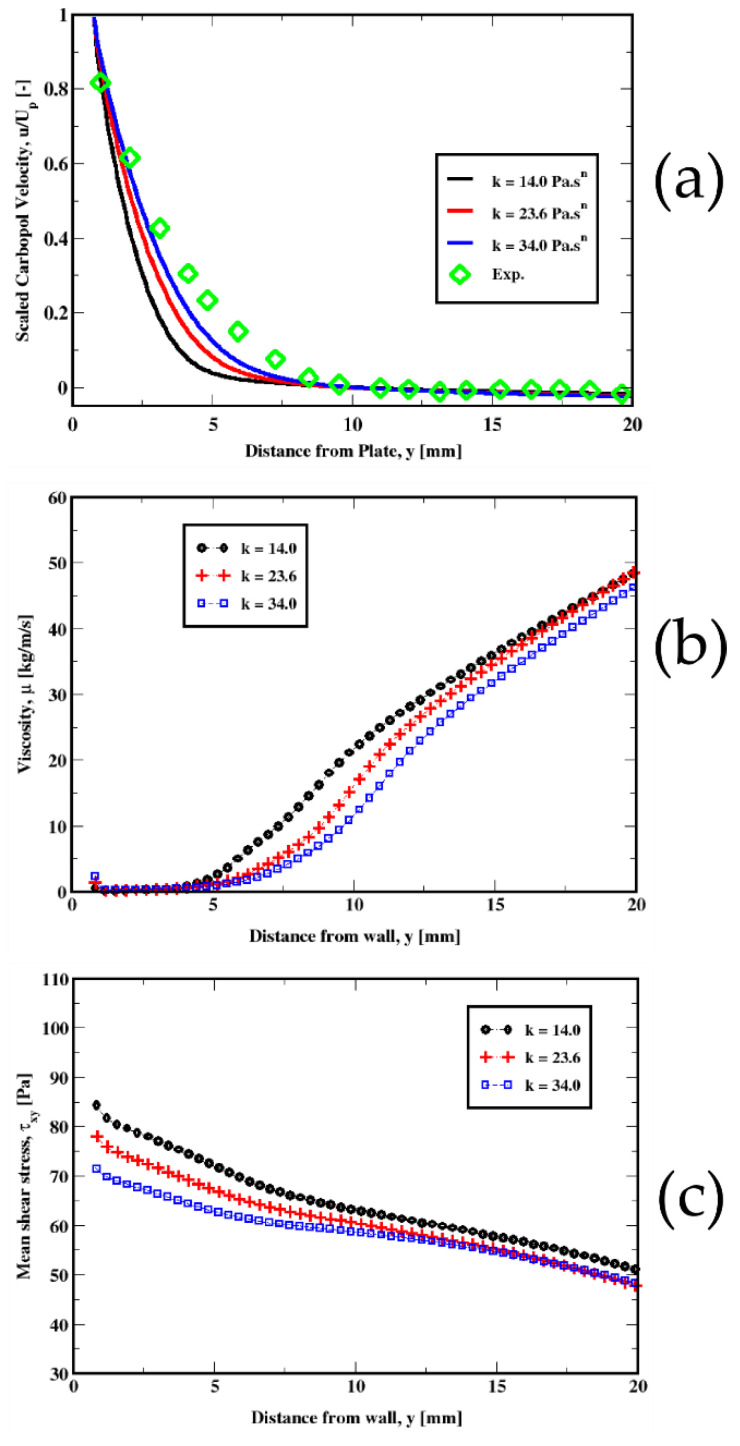
Influence of the consistency index on the distribution of the velocity of the Carbopol fluid (top plot) (**a**) for the plate immersion velocity of 1 mm/s. The middle (**b**) and the bottom (**c**) plots represent the variation in the kinematic viscosity and the mean shear stress, respectively.

In Figure 11, Figure 12, Figure 13 and Figure 14, we plot the variations in the mean velocity (U), the kinematic viscosity (μ/ρ), and the mean shear stress ($\tau_{xy}$) for different material properties.

Figure 11a compares the velocity predictions for different values of the yield stress. For smaller values of the yield stress, we see a better agreement with the experimental data. We also notice similar behavior for the viscosity and the mean shear stress shown in Figure 11b,c. The tendency of the fluid to resist shearing motion as conveyed by the viscosity, clearly increases with the yield stress as shown in Figure 11b. This plot further exhibits a steeper increase in the viscosity within the transition region between the fluid-like and the solid-like behaviors. Figure 11c also shows how the mean shear stress changes with the tangential shearing forces between the fluid and the plate, from near the plate to the far-field regions. These clearly indicate an increase in the friction at the plate with the yield stress, which result in slower velocities.

For different values of the power-law exponent (n), we could see similar tendencies (see Figure 12). However, the variations are not that accentuated as is the case for the yield stress discussed above. Only slight increases are apparent in both the viscosity and the mean shear stress (see Figure 12b,c), which result in a slight change in the velocity, especially for n = 0.25, where the mean shear stress is relatively higher compared to n = 0.38 and n = 0.50. The viscous and the apparent viscosities plotted in Figure 13 show that the apparent viscosity is one order of magnitude larger than the regular (shear) viscosity for each of the power-law exponents used in this study. Therefore, even though some shear-thinning effects takes place, the yield stress effects appear to be dominant. Piau & Debiane [36] and later Boujlel et al. [1] showed that the slope is determined by the power law exponent. In other words, the shear-thinning effects strongly influence the velocity field in the boundary layer.

The velocity distribution in the vicinity of the plate plotted in Figure 14, shows that an increase in the consistency index results in a steeper profile, thus reducing the discrepancy. Typically, the change in the consistency index appears to show a similar change in the viscosity and the mean shear stress. This is illustrated by an increase in the viscosity and in the mean shear stress, especially in the wall region (see Figure 14b,c), causing a flatter profile as a result of the increased friction at the wall.

## 7. Conclusions

The boundary layer experiments of Boujlel et al. [1], where a plate is slowly immersed into a Carbopol yield-stress fluid, are numerically investigated. The fluid is modeled as a Herschel–Bulkley fluid. The numerical approach uses the Papanastasiou regularization of the apparent viscosity associated with the Herschel–Bulkley constitutive model in the context of the overset meshing technique. The physics of the flow appears to be adequately captured for the three flow conditions investigated in this work. It is seen that the boundary layer develops in the vicinity of the plate as the leading edge steadily advances into the fluid. The velocity distribution seems to be in good agreement with the measurements. Specifically, the upward velocity distributions of the unyielded material are reasonably captured. However, a slight mismatch is noticeable within the yielded region near the solid-plug flow. It is shown that the transition between the fluid-like and the solid-like states, is very sensitive to the rheological properties of the fluid. Subsequent simulations show a strong dependence of the mean shear stress on these rheological properties that could cause different values for the friction at the wall. This behavior is consistent with the existing theories [36]. In our numerical investigations, we assume a no-slip condition at the walls. The influence of the slip at the walls (see [36,38]) is left for future work.

## Figures and Tables

**Table 1:** Flow conditions and the associated dimensionless numbers.

Case	$U_{p}\;[m/s]$	Re [-]	Bi [-]
1	1	5.2 × 10^−5^	2.93
2	3	3.1 × 10^−4^	1.93
3	5	7.0 × 10^−4^	1.59

**Table 2:** Summary of the mesh densities.

Mesh Details		Coarse	Medium	Finer
Wall-normal [mm]	$\Delta y_{\min}$	0.2	0.15	0.1
	$\Delta y_{\max}$	0.4	0.3	0.2
Streamwise, Δx [mm]		1.25	1	0.8
Mesh size		60,404	89,940	138,824

**Table 3:** Summary of the boundary layer thickness sensitivities to the mesh refinement and the regularization model.

Experiments [mm]	Simulations					
9.01 ± 0.20	Mesh Sensitivity [mm]			Regularization Sensitivity [mm]		
	Coarse	Medium	Fine	Simple	Bercovier–Engelman	Papanastasiou
	8.64	8.92	9.33	12.67	9.21	8.92

**Table 4:** Different rheological properties. (*) Bi is calculated for the plate immersion velocity of 1 mm/s. (-) refers to the same rheological property as the “Baseline” measured property.

	$\tau_{0}\;[\mathrm{Pa}]$	k	n	^(^*^)^ Bi [-]
Baseline	59.5	23.6	0.38	2.94
Yield stress	40.0	-	-	1.98
	80.0	-	-	3.95
Consistency	-	14.0	-	4.96
	-	34.0	-	2.04
Power-law exponent	-	-	0.25	2.79
	-	-	0.50	3.09

## Disclaimer

This project was funded by the United States Department of Energy, National Energy Technology Laboratory, in part, through a site support contract. Neither the United States Government nor any agency thereof, nor any of their employees, nor the support contractor, nor any of their employees, makes any warranty, express or implied, or assumes any legal liability or responsibility for the accuracy, completeness, or usefulness of any information, apparatus, product, or process disclosed, or represents that its use would not infringe privately owned rights. Reference herein to any specific commercial product, process, or service by trade name, trademark, manufacturer, or otherwise does not necessarily constitute or imply its endorsement, recommendation, or favoring by the United States Government or any agency thereof. The views and opinions of authors expressed herein do not necessarily state or reflect those of the United States Government or any agency thereof.

## Appendix A

In this Appendix, we present the dimensionless form of the governing equations. Assuming a reference length scale d and a reference velocity $U_{p}$, we can define dimensionless length, time, velocity, and pressure as:

(A1) $$x^{*}=x/d,\;\;\;t^{*}=\eta_{0}t/\rho d^{2},\;\;\;v^{*}=v/U_{p},\;\;\;p^{*}=pd/\eta_{0}U_{p}$$

where $\eta_{0}=k{\left(U_{p}/d\right)}^{n-1}$ is a characteristic viscosity associated with Herschel–Bulkley fluids. Thus, the dimensionless mass and momentum equations can be written as:

(A2) $${\mathrm{div}}^{*}v^{*}=0$$

(A3) $$\frac{\partial v^{*}}{\partial t^{*}}+Re\;[{\mathrm{grad}}^{*}v^{*}]v^{*}=-{\mathrm{grad}}^{*}p^{*}+\mathrm{div}\left[\left(1+Bi\right)A_{1}^{*}\right]+b^{*}$$

where $b^{*}=\left(\rho e^{2}/\eta_{0}U_{p}\right)b$ is the dimensionless body force and Re and Bi are the Reynolds and Bingham numbers, respectively. In the limit of Re→0, the inertia does not contribute to the flow dynamics.

## References

[1] Boujlel J., Maillard M., Lindner A., Ovarlez G., Chateau X., Coussot P. (2012). Boundary layer in pastes—Displacement of a long object through a yield stress fluid. J. Rheol..

[2] Schowalter E.R. (1978). Mechanics of Non-Newtonian Fluids.

[3] Oldroyd J.G. (1984). An approach to non-Newtonian fluid mechanics. J. Non-Newton. Fluid Mech..

[4] Larson R.G. (1999). The Structure and Rheology of Complex Fluids.

[5] Li M.-G., Feng F., Wu W.-T., Massoudi M. (2020). Numerical Simulations of the Flow of a Dense Suspension Exhibiting Yield-Stress and Shear-Thinning Effects. Energies.

[6] Fernandes C., Fakhari A., Tukovic Ž. (2021). Non-Isothermal Free-Surface Viscous Flow of Polymer Melts in Pipe Extrusion Using an Open-Source Interface Tracking Finite Volume Method. Polymers.

[7] Wong L.S. (2022). Durability Performance of Geopolymer Concrete: A Review. Polymers.

[8] Batra R.C. (2006). Elements of Continuum Mechanics.

[9] Haupt P. (2002). Continuum Mechanics and Theory of Materials.

[10] Liu I.S. (2002). Continuum Mechanics.

[11] Massoudi M., Vaidya A. (2008). On some generalizations of the second grade fluid model. Nonlinear Anal. Part II Real World Appl..

[12] Rivlin R.S., Ericksen J.L. (1955). Stress deformation relations for isotropic materials. J. Rat. Mech. Anal..

[13] Truesdell C., Noll W. (1992). The Non-Linear Field Theories of Mechanics.

[14] Deshpande A.P., Krishnan J.M., Sunil P.B. (2010). Rheology of Complex Fluids.

[15] Denn M.M., Bonn D. (2011). Issues in the flow of yield-stress liquids. Rheol. Acta.

[16] Beris A.N., Tsamopoulos J.A., Armstrong R.C., Brown R.A. (1985). Creeping motion of a sphere through a Bingham plastic. J. Fluid Mech..

[17] Lootens D., Hébraud P., Lécolier E., Van Damme H. (2004). Gelation, Shear-Thinning and Shear-Thickening in Cement Slurries. Oil Gas Sci. Technol..

[18] Banfill P.F. The rheology of fresh cement and concrete-a review. Proceedings of the 11th International Cement Chemistry Congress.

[19] Tao C., Rosenbaum E., Kutchko B.G., Massoudi M. (2021). A Brief Review of Gas Migration in Oilwell Cement Slurries. Energies.

[20] Carreau P.J., De Kee D., Chhabra R.J. (1997). Rheology of Polymeric System.

[21] Tao C., Kutchko B.G., Rosenbaum E., Wu W.-T., Massoudi M. (2019). Steady Flow of a Cement Slurry. Energies.

[22] Mewis J., Spaull A.J.B. (1976). Rheology of Concentrated Dispersions. Adv. Colloid Interface Sci..

[23] Mewis J., Wagner N.J. (2012). Colloidal Suspension Rheology.

[24] Agassant J.F., Avenas P., Carreau P.J., Vergnes B., Vincent M. (2017). Polymer Processing: Principles and Modeling.

[25] Denn M.M. (2009). Simulation of polymer melt processing. AIChE J..

[26] Middleman S. (1977). Fundamentals of Polymer Processing.

[27] Ziaee H., Arabloo M., Ghazanfari M.H., Rashtchian D. (2015). Herschel–Bulkley rheological parameters of lightweight colloidal gas aphron (CGA) based fluids. Chem. Eng. Res. Des..

[28] Wang S., Yuan C., Zhang C., Chen L., Liu J. (2016). Rheological properties with temperature response characteristics and a mechanism of solid-free polymer drilling fluid at low temperatures. Appl. Sci..

[29] Shafiei M., Bryant S., Balhoff M., Huh C., Bonnecaze R.T. (2017). Hydrogel formulation for sealing cracked wellbores for CO_2_ storage. Appl. Rheol..

[30] Chauhan G., Verma A., Das A., Ojha K. (2018). Rheological studies and optimization of Herschel-Bulkley flow parameters of viscous karaya polymer suspensions using GA and PSO algorithms. Rheol. Acta.

[31] Zheng W., Wu X., Huang Y. (2020). Impact of polymer addition, electrolyte, clay and antioxidant on rheological properties of polymer fluid at high temperature and high pressure. J. Pet. Explor. Prod. Technol..

[32] Milián D., Roux D.C., Caton F., El Kissi N. (2022). Rheological behavior of gel polymer electrolytes: Yield stress and viscoelasticity. Rheol. Acta.

[33] Oldroyd J.G. (1947). Two-dimensional plastic flow of a Bingham solid. A plastic boundary-layer theory for slow motion. Proc. Camb. Philos. Society. Math. Phys. Sci..

[34] Piau J.-M. (2002). Viscoplastic boundary layer. J. Non-Newton. Fluid Mech..

[35] Balmforth N.J., Craster R.V., Hewitt D.R. (2021). Building on Oldroyd’s viscoplastic legacy: Perspectives and new developments. J. Non-Newton. Fluid Mech..

[36] Piau J.-M., Debiane K. (2004). The adhesive or slippery flat plate viscoplastic boundary layer for a shear-thinning power-law viscosity. J. Non-Newton. Fluid Mech..

[37] Ahonguio F., Jossic L., Magnin A. (2016). Influence of slip on the flow of a yield stress fluid around a flat plate. AIChE J..

[38] Ahonguio F., Jossic L., Magnin A., Dufour F. (2016). Flow of an elasto-viscoplastic fluid around a flate plate: Experimental and numerical data. J. Non-Newton. Fluid Mech..

[39] Balmforth N.J., Craster R.V., Hewitt D.R., Hormozi S., Maleki A. (2017). Viscoplastic boundary layers. J. Fluid Mech..

[40] Chevalier T., Rodts S., Chateau X., Boujlel J., Maillard M., Coussot P. (2013). Boundary layer (shear-band) in frustrated viscoplastic flows. EPL.

[41] Herschel W.H., Bulkley R. (1926). Measurement of Consistency as Applied to Rubber-Benzene Solutions. Am. Soc. Test Proc..

[42] Headrick E.D., Spaulding R., Rosenbaum E., Kutchko B., Massoudi M. (2022). The Effects of Conditioning and Additives on the Viscosity Measurement of Cement Slurries.

[43] Slattery J.C. (1999). Advanced Transport Phenomena.

[44] Barnes H.A. (1999). The yield stress—A review or ‘παντα ρει’—Everything flows?. J. Non-Newton. Fluid Mech..

[45] Barnes H.A. (2007). The ‘yield stress myth?’ paper–21 years on. Appl. Rheol..

[46] Barnes H.A., Walters K. (1985). The yield stress myth?. Rheol. Acta.

[47] Assaad J.J., Harb J., Maalouf Y. (2014). Measurement of Yield Stress of Cement Pastes Using the Direct Shear Test. J. Non-Newton. Fluid Mech..

[48] Assaad J.J., Harb J., Maalouf Y. (2016). Effect of Vane Configuration on Yield Stress Measurements of Cement Pastes. J. Non-Newton. Fluid Mech..

[49] Watts B., Tao C., Ferraro C., Masters F. (2018). Proficiency analysis of VCCTL results for heat of hydration and mortar cube strength. Constr. Build. Mater..

[50] Moller P., Fall A., Chikkadi V., Derks D., Bonn D. (2009). An Attempt to Categorize Yield Stress Fluid Behaviour. Philos. Trans. R. Soc. A Math. Phys. Eng. Sci..

[51] Dinkgreve M., Paredes J., Denn M.M., Bonn D. (2016). On Different Ways of Measuring ‘the’ Yield Stress. J. Non-Newton. Fluid Mech..

[52] Nguyen Q.D., Boger D.V. (1992). Measuring the Flow Properties of Yield Stress Fluids. Annu. Rev. Fluid Mech..

[53] Coussot P. (2014). Yield Stress Fluid Flows: A Review of Experimental Data. J. Non-Newton. Fluid Mech..

[54] Coussot P., Nguyen Q.D., Huynh H.T., Bonn D. (2002). Viscosity Bifurcation in Thixotropic, Yielding Fluids. J. Rheol..

[55] Tao C., Rosenbaum E., Kutchko B., Massoudi M. (2020). The Importance of Vane Configuration on Yield Stress Measurements of Cement Slurry.

[56] Saak A.W., Jennings H.M., Shah S.P. (2001). The Influence of Wall Slip on Yield Stress and Viscoelastic Measurements of Cement Paste. Cem. Concr. Res..

[57] Barnes H.A. (1995). A Review of the Slip (Wall Depletion) of Polymer Solutions, Emulsions and Particle Suspensions in Viscometers: Its Cause, Character, and Cure. J. Non-Newton. Fluid Mech..

[58] Nguyen Q.D., Boger D.V. (1985). Direct Yield Stress Measurement with the Vane Method. J. Rheol..

[59] Liddel P.V., Boger D.V. (1996). Yield Stress Measurements with the Vane. J. Non-Newton. Fluid Mech..

[60] Banfill P.F.G., Saunders D.C. (1981). On the Viscometric Examination of Cement Pastes. Cem. Concr. Res..

[61] Nguyen Q.D., Boger D.V. (1983). Yield Stress Measurement for Concentrated Suspensions. J. Rheol..

[62] Tao C., Kutchko B.G., Rosenbaum E., Massoudi M. (2020). A Review of Rheological Modeling of Cement Slurry in Oil Well Applications. Energies.

[63] Krieger I.M., Dougherty T.J. (1959). A mechanism for non-Newtonian flow in suspensions of rigid spheres. Trans. Soc. Rheol..

[64] Bingham E.C. (1922). Fluidity and Plasticity.

[65] Denn M.M. (2008). Polymer Melt Processing: Foundations in Fluid Mechanics and Heat Transfer.

[66] Macosko C.W. (1994). Rheology: Principles, Measurements, and Applications.

[67] OpenFOAM v2012. http://www.openfoam.org.

[68] Papanastasiou T.C. (1987). Flows of materials with yield. J. Rheol..

[69] Allouche M., Frigaard I.A., Sona G. (2000). Static wall layers in the displacement of two viscoplastic fluids in a plane channel. J. Fluid Mech..

[70] Bercovier M., Engelman M. (1980). A finite-element method for incompressible non-Newtonian flows. J. Comput. Phys..

[71] Frigaard I.A., Nouar C. (2005). On the usage of viscosity regularisation methods for visco-plastic fluid flow computation. J. Non-Newtonian Fluid Mech..

[72] Saramito P., Wachs A. (2017). Progress in numerical simulation of yield stress fluid flows. Rheol. Acta.

[73] Putz A.M.V., Burghelea T.I. (2009). The solid–fluid transition in a yield stress shear thinning physical gel. Rheol. Acta.

[74] Piau J.-M. (2007). Carbopol gels: Elastoviscoplastic and slippery glasses made of individual swollen sponges meso- and macroscopic properties, constitutive equations and scaling laws. J. Non-Newton. Fluid Mech..

